# Supplementary material for: Gut Microbiome Studies in Livestock: Achievements, Challenges, and Perspectives
Source: Animals (Basel). 2022 Nov 30;12(23):3375. doi: 10.3390/ani12233375 (PMC9736591; doi:10.3390/ani12233375)
Supplement: Supplementary file 1 [file animals-12-03375-s001.zip › Table S1 ruminants 29112022.pdf]

Table S1: List of most abundant eubacterial genera found across the GIT of ruminant livestock species. Cow, sheep and goat pictures were downloaded from https://commons.wikimedia.org/, https://www.iconspng.com/ and https://freepngdesign.com/, respectively.

	Phylum	Class	Order	Family	Genus	Reference
**Cattle**	**Actinobacteria**	**Actinomycetia**	**Propionibacteriales**	**Propionibacteriaceae**	***Propionibacterium***	**66**
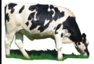		**Coriobacteriia**	**Coriobacteriales**	**Atopobiaceae**	***Atopobium***	**51**
					Olsenella	**59**
			**Eggerthellales**	**Eggerthellaceae**	***Adlercreutzia***	**51**
	**Bacteroidetes**	**Bacteroidia**	**Bacteroidales**	**Bacteroidaceae**	***Bacteroides***	**63,78**
				**Rikenellaceae**	***Alistipes***	**60,78**
				**Prevotellaceae**	***Prevotella***	**46,49,51,55,56,58,59,60,62,63,64,65,66,78**
				**Tannerellaceae**	***Parabacteroides***	**66**
	**Fibrobacteres**	**Fibrobacteres**	**Fibrobacterales**	**Fibrobacteraceae**	***Fibrobacter***	**46,55,58,59,62,78**
	**Firmicutes**	**Bacilli**	**Lactobacillales**	**Lactobacillaceae**	***Lactobacillus***	**56,66**
				**Streptococcaceae**	***Streptococcus***	**62,63**
		**Clostridia**	**Eubacteriales**	**Clostridiacea**	***Clostridium***	**51,65,66,78**
				**Eubacteriaceae**	***Eubacterium***	**51**
				**Lachnospiraceae**	**Acetitomaculum**	**46**
					Blautia	**51,66**
					Butyrivibrio	**46,51,55,60,66**
					Coprococcus	**51,66**
					Dorea	**66**
					Lachnobacterium	**51**
					Lachnospira	**51**
					Moryella	**51**
					Pseudobutyrivibrio	**51**
					Roseburia	**51,58**
					Shuttleworthia	**51**
				**Eubacteriales Family XIII. Incertae Sedis**	***Mogibacterium***	**46,66**
				**Oscillospiraceae**	***Oscillospira***	**59,66**
					Ruminococcus	**46,49,51,55,58,59,62,65,66,78**
		**Erysipelotrichia**	**Erysipelotrichales**	**Coprobacillaceae**	***Sharpea***	**59**
				**Erysipelotrichaceae**	***Bulleidia***	**51,66**
				**Turicibacteraceae**	***Turicibacter***	**66**

Table S1: List of most abundant eubacterial genera found across the GIT of ruminant livestock species. Cow, sheep and goat pictures were downloaded from https://commons.wikimedia.org/, https://www.iconspng.com/ and https://freepngdesign.com/, respectively.

		Negativicutes	Acidaminococcales	Acidaminococcaceae	*Acidaminococcus*	56
					Succiniclasticum	46,51
			Selenomonadales	Selenomonadaceae	*Anaerovibrio*	46
					Mitsuokella	51,56,66
					Selenomonas	51,56,59,62
			Veillonales	Veillonellaceae	*Dialister*	56,66
	Proteobacteria	Alphaproteobacteria	Rhodospirillales	Acetobacteraceae	*Acetobacter*	46
		Deltaproteobacteria	Desulfovibrionales	Desulfovibrionaceae	*Desulfovibrio*	51
			Desulfobacterales	Desulfobulbaceae	*Desulfobulbus*	46
		Gammaproteobacteria	Pseudomonadales	Moraxellaceae	*Acinetobacter*	46
			Aeromonadales	Succinivibrionaceae	*Succinivibrio*	59,62,66
	Spirochaetes	Spirochaetes	Spirochaetales	Spirochaetaceae	*Treponema*	46,55
	Tenericutes	Mollicutes	Anaeroplasmatales	Anaeroplasmataceae	*Acholeplasma*	58
			Mycoplasmatales	Mycoplasmataceae	*Mycoplasma*	46
Sheep 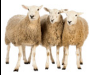	Actinobacteria	Coriobacteriia	Coriobacteriales	Atopobiaceae	*Olsenella*	67
	Bacteroidetes	Bacteroidia	Bacteroidales	Bacteroidaceae	*Bacteroides*	70,75,77,78,79,82
			Flavobacteriales	Flavobacteriaceae	*Flavobacterium*	79
				Rikenellaceae	*Alistipes*	78
				Porphyromonadaceae	*Porphyromonas*	82
				Prevotellaceae	*Alloprevotella*	82
					Prevotella	67-69,74,77-79
				Tannerellaceae	*Parabacteroides*	77
	Fibrobacteres	Fibrobacteres	Fibrobacterales	Fibrobacteraceae	*Fibrobacter*	68,76,78,79,82
	Firmicutes	Bacilli	Bacillales	Bacillaceae	*Bacillus*	79
			Lactobacillales	Lactobacillaceae	*Lactobacillus*	82
				Streptococcaceae	*Lactococcus*	77
					Streptococcus	82
		Clostridia	Eubacteriales	Clostridiaceae	*Clostridium*	70,77-79
				Eubacteriaceae	*Eubacterium*	69
				Lachnospiraceae	*Butyrivibrio*	77,82

Table S1: List of most abundant eubacterial genera found across the GIT of ruminant livestock species. Cow, sheep and goat pictures were downloaded from https://commons.wikimedia.org/, https://www.iconspng.com/ and https://freepngdesign.com/, respectively.

					Catonella	68
					Coprococcus	69,77
					Dorea	74
					Lachnoclostridium	69,82
					Moryella	69
					Oribacterium	67
					Roseburia	68
					Shuttleworthia	67
					Syntrophococcus	67
					Tyzzerella	69
				Eubacteriales Family XIII. Incertae Sedis	*Mogibacterium*	77
				Oscillospiraceae	*Oscillospira*	77,79
					Faecalibacterium	77
					Saccharofermentans	76
				Ruminococcaceae	*Ruminococcus*	67,77,78,82
		Erysipelotrichia	Erysipelotrichales	Erysipelotrichaceae	*Bulleidia*	77
					Anaerorhabdus	79
		Negativicutes	Acidaminococcales	Acidaminococcaceae	*Acidaminococcus*	68
					Succiniclasticum	67- 69,82
			Selenomonadales	Selenomonadaceae	*Anaerovibrio*	82
					Selenomonas	67,71
				Sporomusaceae	*Anaerosinus*	79
			Veillonales	Veillonellaceae	*Dialister*	68
					Veillonella	69
	Fusobacteria	Fusobacteriia	Fusobacteriales	Fusobacteriaceae	*Fusobacterium*	82
				Leptotrichiaceae	*Streptobacillus*	79
	Proteobacteria	Alphaproteobacteria	Caulobacterales	Caulobacteraceae	*Nitrobacteria*	79
			Sphingomonadales	Sphingomonadaceae	*Sphingomonas*	82
		Betaproteobacteria	Burkholderiales	Alcaligenaceae	*Bordetella*	79
			Neisseriales	Neisseriaceae	*Alysiella*	82
		Deltaproteobacteria	Desulfovibrionales	Desulfovibrionaceae	*Desulfovibrio*	75,77
		Epsilonproteobacteria	Campylobacterales	Campylobacteraceae	*Campylobacter*	75

Table S1: List of most abundant eubacterial genera found across the GIT of ruminant livestock species. Cow, sheep and goat pictures were downloaded from https://commons.wikimedia.org/, https://www.iconspng.com/ and https://freepngdesign.com/, respectively.

		Gammaproteobacteria	Aeromonadales	Succinivibrionaceae	*Moraxella*	82
					Ruminobacter	77,82
					Succinivibrio	74,76
			Alteromonadales	Shewanellaceae	*Shewanella*	82
			Enterobacterales	Enterobacteriaceae	*Escherichia*	69
					Shigella	69
			Oceanospirillales	Halomonadaceae	*Halomonas*	82
			Pasteurellales	Pasteurellaceae	*Bibersteinia*	82
					Mannheimia	82
			Pseudomonadales	Moraxellaceae	*Acinetobacter*	79
					Alkanindiges	79
					Psychrobacter	79
				Pseudomonadaceae	*Pseudomonas*	79
	Spirochaetes	Spirochaetes	Spirochaetales	Spirochaetaceae	*Treponema*	68,74,82
	Tenericutes	Mollicutes	Acholeplasmatales	Acholeplasmataceae	*Acholeplasma*	79
	Verrucomicrobia	Verrucomicrobiae	Verrucomicrobiales	Akkermansiaceae	*Akkermansia*	75
Goat 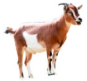	Actinobacteria	Actinomycetia	Bifidobacteriales	Bifidobacteriaceae	*Bifidobacterium*	100
		Coriobacteriia	Coriobacteriales	Atopobiaceae	*Olsenella*	67,100
	Bacteroidetes	Bacteroidia	Bacteroidales	Bacteroidaceae	*Bacteroides*	78,90,100
				Rikenellaceae	*Alistipes*	78
				Prevotellaceae	*Alloprevotella*	93
					Prevotella	67,78,84,85,90,91,96,98
	Elusimicrobia	Elusimicrobia	Elusimicrobiales	Elusimicrobiaceae	*Elusimicrobium*	100
	Fibrobacteres	Fibrobacteres	Fibrobacterales	Fibrobacteraceae	*Fibrobacter*	78,84,93,96,98
	Firmicutes	Bacilli	Bacillales	Bacillaceae	*Bacillus*	88
				Bacillales Family XII. Incertae Sedis	*Exiguobacterium*	88
			Lactobacillales	Lactobacillaceae	*Lactobacillus*	88,100
				Streptococcaceae	*Streptococcus*	100
		Clostridia	Eubacteriales	Clostridiaceae	*Butyricicoccus*	90
					Clostridium	78,84,88,96,98,100
				Eubacteriaceae	*Eubacterium*	93,98

Table S1: List of most abundant eubacterial genera found across the GIT of ruminant livestock species. Cow, sheep and goat pictures were downloaded from https://commons.wikimedia.org/, https://www.iconspng.com/ and https://freepngdesign.com/, respectively.

				Eubacteriales Family XIII. Incertae Sedis	*Anaerovorax*	98
					Mogibacterium	98
				Lachnospiraceae	*Butyrivibrio*	84,85
					Coprococcus	84,93
					Lachnoclostridium	88,93,98
					Oribacterium	67
					Pseudobutyrivibrio	84
					Shuttleworthia	67
					Stomatobaculum	100
					Syntrophococcus	67
				Oscillospiraceae	*Oscillospira*	84,96
					Faecalibacterium	88
					Papillibacter	98
					Sporobacter	79
				Ruminococcaceae	*Ruminococcus*	67,78,84,91,96,98,100
		Negativicutes	Acidaminococcales	Acidaminococcaceae	*Succiniclasticum*	84,85,93,100
			Selenomonadales	Selenomonadaceae	*Selenomonas*	67,84,91,98
	Lenthispherae	Lentisphaeria	Victivallales	Victivallaceae	*Victivallis*	98
	Proteobacteria	Betaproteobacteria	Burkholderiales	Comamonadaceae	*Variovorax*	88
			Neisseriales	Neisseriaceae	*Bergeriella*	90
		Deltaproteobacteria	Desulfovibrionales	Desulfovibrionaceae	*Desulfovibrio*	96,98
		Epsilonproteobacteria	Campylobacterales	Campylobacteraceae	*Campylobacter*	100
		Gammaproteobacteria	Aeromonadales	Succinivibrionaceae	*Succinivibrio*	84,91
					Moraxella	100
			Enterobacterales	Enterobacteriaceae	*Escherichia*	88
					Shigella	88
			Pasteurellales	Pasteurellaceae	*Biberstenia*	90
					Mannheimia	90
			Xanthomonadales	Xanthomonadaceae	*Stenotrophomonas*	88
	Spirochaetes	Spirochaetes	Spirochaetales	Spirochaetaceae	*Treponema*	84,98
