# Supplementary material for: Gut Microbiome Studies in Livestock: Achievements, Challenges, and Perspectives
Source: Animals (Basel). 2022 Nov 30;12(23):3375. doi: 10.3390/ani12233375 (PMC9736591; doi:10.3390/ani12233375)
Supplement: Supplementary file 1 [file animals-12-03375-s001.zip › Table S2 non-ruminants 29112022.pdf]

Table S2: List of most abundant eubacterial genera found across the GIT of monogastric livestock species. The pig and chicken pictures were freely downloaded from https://www.freepik.com/ and https://pnghut.com/, respectively.

	Phylum	Class	Order	Family	Genus	Reference
Pig 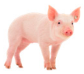	Actinobacteria	Actinomycetia	Bifidobacteriales	Bifidobacteriaceae	*Bifidobacterum*	106,113,125
			Corynebacteriales	Corynebacteriaceae	*Corynebacterium*	113
		Coriobacteriia	Coriobacteriales	Atopobiaceae	*Olsenella*	107,113,127
				Coriobacteriaceae	*Collinsella*	106,113,116,127
	Bacteroidetes	Bacteroidia	Bacteroidales	Bacteroidaceae	*Bacteroides*	106,110,113,116,120,127
				Rikenellaceae	*Alistipes*	113,116,126
				Prevotellaceae	*Alloprevotella*	112,116,126
					Prevotella	106,109,110,112,113,116,120,125-128
					Parabacteroides	113,120
			Flavobacteriales	Flavobacteriaceae	*Flavobacterium*	106,113
	Fibrobacteres	Fibrobacteres	Fibrobacterales	Fibrobacteraceae	*Fibrobacter*	113,127
	Firmicutes	Bacilli	Bacillales	Bacillaceae	*Bacillus*	106,113
				Planococcaceae	*Rummeliibacillus*	113,127
			Lactobacillales	Enterococcaceae	*Enterococcus*	113,127
				Lactobacillaceae	*Lactobacillus*	106-110,113,116,120,125-128
					Pediococcus	113
					Weissella	113
				Streptococcaceae	*Lactococcus*	113
					Streptococcus	106,107,109,113,126-128
		Clostridia	Eubacteriales	Christensenellacaceae		110,126
				Clostridiacea	*Clostridium*	106,107,109,110,113,116,120,126-128
				Eubacteriaceae	*Eubacterium*	113,116
				Lachnospiraceae	*Agathobacter*	112
					Blautia	110,113,116,127
					Butyrivibrio	112,113
					Coprococcus	110,113,116,128
					Dorea	110,113

Table S2: List of most abundant eubacterial genera found across the GIT of monogastric livestock species. The pig and chicken pictures were freely downloaded from https://www.freepik.com/ and https://pnghut.com/, respectively.

					Oribacterium	113
					Pseudobutyrivibrio	113,116
					Roseburia	110,113,116
					Syntrophococcus	113
				Mogibacteriaceae	*Mogibacterium*	113
				Oscillospiraceae	*Faecalibacterium*	110,113,116,120,127
					Oscillospira	110,113,128
					Papillibacter	113
					Ruminiclostridium	112,116
					Subdoligranulum	113,116
				Peptococcaceae	*Peptococcus*	106,113
				Peptostreptococcaceae	*Romboutsia*	107,126
					Sporacetigenium	113
					Terrisporobacter	126
				Ruminococcaceae	*Ruminococcus*	106,109,113,120,126-128
		Erysipelotrichia	Erysipelotrichales	Coprobacillaceae	*Sharpea*	113
				Erysipelotricaceae	*Catenibacterium*	113,116
					Holdemania	113,128
					Solobacterium	106,112,116
				Turicibacteraceae	*Turicibacter*	107,113,126
		Negativicutes	Acidaminococcales	Acidaminococcaceae	*Acidaminococcus*	113,116
					Phascolarctobacterium	106,110,113,116,127
			Selenomonadales	Selenomonadaceae	*Anaerovibrio*	110,113,116,127
					Mitsuokella	113
					Selenomonas	113
			Veillonales	Veillonellaceae	*Megasphera*	106,113,116,127
					Veillonella	113,116
				Thermohalobacteraceae		106

Table S2: List of most abundant eubacterial genera found across the GIT of monogastric livestock species. The pig and chicken pictures were freely downloaded from https://www.freepik.com/ and https://pnghut.com/, respectively.

	**Fusobacteria**	**Fusobacteriia**	**Fusobacteriales**	**Fusobacteriaceae**	***Fusobacterium***	**106,116,127**
	**Proteobacteria**	**Alphaproteobacteria**	**Hyphomicrobiales**	**Brucellacea**	***Ochrobactrum***	**113**
		**Betaproteobacteria**	**Burkholderiales**	**Burkholderiaceae**	***Ralstonia***	**112**
		**Deltaproteobacteria**	**Desulfovibrionales**	**Desulfovibrionaceae**	***Desulfovibrio***	**110,113,126,127**
					Lawsonia	**107,113**
		**Epsilonproteobacteria**	**Campylobacterales**	**Helicobacteraceae**	***Helicobacter***	**107,110,113,116**
		**Gammaproteobacteria**	**Aeromonadales**	**Succinivibrionaceae**	***Succinivibrio***	**113,127**
			**Enterobacterales**	**Enterobacteriaceae**	***Escherichia***	**106,107,110,113,116,125-127**
					Shigella	**107,110,113,116,126**
			**Pasteurellales**	**Pasteurellaceae**	***Actinobacillus***	**107-109,113**
			**Pseudomonadales**	**Moraxellaceae**	***Acinetobacter***	**113**
			**Xanthomonadales**	**Xanthomonadaceae**	***Stenotrophomonas***	**113,126**
		**Epsilonproteobacteria**	**Campylobacterales**	**Campylobacteraceae**	***Campylobacter***	**106,109,110,113**
	**Spirochaetes**	**Spirochaetes**	**Spirochaetales**	**Spirochaetaceae**	***Treponema***	**113,126-128**
**Chicken** 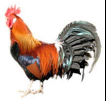	**Actinobacteria**	**Actinomycetia**	**Actinomycetales**	**Actinomycetaceae**	***Actinomyces***	**141**
			**Coriobacteriales**	**Atopobiaceae**	***Olsenella***	**144**
				**Coriobacteriaceae**	***Collinsella***	**134,136**
					Enorma	**136**
					Gordonibacter	**136**
			**Corynebacteriales**	**Corynebacteriaceae**	***Corynebacterium***	**133,149,159**
			**Eggerthellales**	**Eggerthellaceae**	***Eggerthella***	**131**
			**Micrococcales**	**Micrococcaceae**	***Arthrobacter***	**134**
					Kocuria	**149**
					Rothia	**149**
	**Bacteroidetes**	**Bacteroidia**	**Bacteroidales**	**Bacteroidaceae**	***Bacteroides***	**131,133,136,137,141,143,144,146-150,159**
					Mediterranea	**136**
				**Barnesiellaceae**	***Barnesiella***	**131,136,144,146**
					Coprobacter	**146,148**

Table S2: List of most abundant eubacterial genera found across the GIT of monogastric livestock species. The pig and chicken pictures were freely downloaded from https://www.freepik.com/ and https://pnghut.com/, respectively.

				**Muribaculaceae**	***Muribaculum***	**136**
				**Odoribacteraceae**	***Odoribacter***	**131,136,148**
					Butyricimonas	**136,146,148**
				**Prevotellaceae**	***Alloprevotella***	**143**
					Parabacteroides	**131,136,144,146,148**
					Prevotella	**144,147**
				**Rikenellaceae**	***Alistipes***	**131,143,144,146,148**
					Rikenella	**148**
		**Flavobacteria**	**Flavobacteriales**	**Flavobacteriaceae**	***Flavobacterium***	**141**
	**Fibrobacteres**	**Fibrobacteres**	**Fibrobacterales**	**Fibrobacteraceae**	***Fibrobacter***	**143**
	**Firmicutes**	**Bacilli**	**Bacillales**	**Bacillaceae**	***Bacillus***	**133,139**
					Lysinibacillus	**139,140**
				**Paenibacillaceae**	***Brevibacillus***	**140**
					Paenibacillus	**140**
				**Planococcaceae**	***Kurthia***	**140**
			**Lactobacillales**	**Carnobacteriaceae**	***Granulicatella***	**134**
				**Enterococcaceae**	***Enterococcus***	**131,134,136,137,139,141,149,150**
				**Lactobacillaceae**	***Lactobacillus***	**131,133,134,136,137,139-141,144,146-149,159**
					Pediococcus	**133**
					Weissella	**149**
				**Streptococcaceae**	***Streptococcus***	**133,134,149**
				**Staphylococcaceae**	***Staphylococcus***	**131**
		**Clostridia**	**Eubacteriales**	**Christensenellacaceae**		**148**
				**Clostridiacea**	***Butyricicoccus***	**131,133,134,136,144**
					Clostridium	**133,134,136,137,147-149,159**
				**Desulfitobacteriaceae**	***Dehalobacter***	**144**
				**Eubacteriaceae**	***Eubacterium***	**133,134,136,147**
					Intestinimonas	**146**

Table S2: List of most abundant eubacterial genera found across the GIT of monogastric livestock species. The pig and chicken pictures were freely downloaded from https://www.freepik.com/ and https://pnghut.com/, respectively.

				**Gracilibacteraceae**	***Gracilibacter***	**144**
				**Lachnospiraceae**	***Anaerostipes***	**148**
					Blautia	**131,133,134,136,144,146,147**
					Butyrivibrio	**133**
					Coprococcus	**131,148**
					Dorea	**131,134,148**
					Lachnoclostridium	**149**
					Roseburia	**131,133,134**
					Shuttleworthia	**146**
					Tyzzerella	**146**
				**Oscillospiraceae**	***Anaerobacterium***	**144**
					Anaerofilum	**136,146**
					Anaeromassilibacillus	**136**
					Anaerotroncus	**131,133,134,136,146,148**
					Drancourtella	**136**
					Ethanoligenens	**133**
					Faecalibacterium	**133,134,136,137,141,146-148,150**
					Flavonifractor	**136,146**
					Oscillibacter	**131,146**
					Oscillospira	**148**
					Papillibacter	**131**
					Pseudoflavonifractor	**134,136**
					Ruminiclostridium	**144,146**
					Ruminococcus	**133,134,144**
					Subdoligranulum	**131,133,134,144,146**
				**Peptostreptococcaceae**	***Romboutsia***	**137,149**
					Terrisporobacter	**137**
		**Erysipelotrichia**	**Erysipelotrichales**	**Coprobacillaceae**	***Coprobacillus***	**141**

Table S2: List of most abundant eubacterial genera found across the GIT of monogastric livestock species. The pig and chicken pictures were freely downloaded from https://www.freepik.com/ and https://pnghut.com/, respectively.

				**Erysipelotrichaceae**	***Faecalicoccus***	**144**
					Erysipelatoclostridium	**144**
					Massilimicrobiota	**136**
					Merdibacter	**146**
				**Turicibacteraceae**	***Turicibacter***	**131,139**
		**Firmicutes sensu stricto incertae sedis**			***Negativibacillus***	**146**
		**Negativicutes**	**Acidaminococcales**	**Acidaminococcaceae**	***Phascolarctobacterium***	**143,146,148**
			**Selenomonadales**	**Selenomonadaceae**	***Anaerovibrio***	**144**
					Megamonas	**136,141,146**
			**Veillonales**	**Veillonellaceae**	***Megasphera***	**136**
	**Fusobacteria**	**Fusobacteriia**	**Fusobacteriales**	**Fusobacteriaceae**	***Fusobacterium***	**134,159**
	**Lenthispherae**	**Lentisphaeria**	**Victivallales**	**Victivallaceae**	***Victivallis***	**146**
	**Proteobacteria**	**Alphaproteobacteria**	**Caulobacterales**	**Caulobacteraceae**	***Brevundimonas***	**141**
			**Rhodobacterales**	**Rhodobacteraceae**	***Rhodobacter***	**141**
		**Betaproteobacteria**	**Burkholderiales**	**Comamonadaceae**	***Comamonas***	**141**
					Delftia	**139**
				**Suetterellaceae**	***Parasutterella***	**131**
					Sutterella	**148**
		**Deltaproteobacteria**	**Desulfovibrionales**	**Desulfovibrionaceae**	***Bilophila***	**146**
					Desulfovibrio	**131,136,143**
		**Epsilonproteobacteria**	**Campylobacterales**	**Helicobacteraceae**	***Helicobacter***	**146-148,159**
		**Gammaproteobacteria**	**Enterobacterales**	**Enterobacteriaceae**	***Escherichia***	**131,136,137,139,146,149**
					Klebsiella	**134**
					Shigella	**134,137,146,149,159**
			**Gammaproteobacteria incertae sedis**		***Endoriftia***	**133**
			**Moraxellales**	**Moraxellaceae**	***Psychrobacter***	**142**
				**Morganellaceae**	***Providencia***	**133**
			**Pasteurellales**	**Pasteurellaceae**	***Gallibacterium***	**141,147,150**

Table S2: List of most abundant eubacterial genera found across the GIT of monogastric livestock species. The pig and chicken pictures were freely downloaded from https://www.freepik.com/ and https://pnghut.com/, respectively.

			**Pseudomonadales**	**Moraxellaceae**	***Acinetobacter***	**134,159**
				**Pseudomonadaceae**	***Pseudomonas***	**141**
	**Synergistetes**	**Synergistia**	**Synergistales**	**Synergistaceae**	***Synergistes***	**143**
	**Tenericutes**	**Mollicutes**	**Acholeplasmatales**	**Acholeplasmataceae**	***Acholeplasma***	**144**
	**Verrucomicrobia**	**Verrucomicrobiae**	**Verrucomicrobiales**	**Akkermansiaceae**	***Akkermansia***	**136,144**
